# Experimental investigation of sophorolipids injection for enhanced oil recovery in carbonate reservoir

**DOI:** 10.3389/fmicb.2026.1895018

**Published:** 2026-07-29

**Authors:** Hala Al-Hemeidi, Sami Al-Khamisi, Yahya Al-Wahaibi, Saif Al-Bahry, Sanket J. Joshi

**Affiliations:** 1 Department of Petroleum and Chemical Engineering, College of Engineering, Sultan Qaboos University, Muscat, Oman; 2 Oil and Gas Research Centre, Sultan Qaboos University, Muscat, Oman; 3 A’Sharqiyah University, Ibra, Oman; 4 Department of Biology, College of Science, Sultan Qaboos University, Muscat, Oman; 5 Amity Institute of Microbial Technology, Amity University Rajasthan, Jaipur, India; 6 Amity Microbial Culture Centre, Amity University Rajasthan, Jaipur, India

**Keywords:** biosurfactant, carbonate reservoir, core flooding, enhanced oil recovery, sophorolipids, wettability alteration

## Abstract

**Inroduction:**

Biosurfactants have become a topic of interest in recent years as low-cost and environmentally friendly agents. Their potential to improve oil recovery has been addressed previously, yet there are some concerns about their performance under reservoir extreme conditions. This study aims to assess Sophorolipids, an anionic biosurfactant, to enhance oil recovery in one of the Omani tight carbonate reservoirs characterized by high temperature and high salinity conditions.

**Methods:**

This was conducted by examining its effect on interfacial tension and wettability of carbonate at different concentrations, followed by core flooding experiments and ending by observing the stability of the biosurfactant at reservoir conditions.

**Results:**

Results showed that the biosurfactant solution at the determined critical micelle concentration (CMC) of 1% was able to decrease the surface tension (ST) from 65.3 ± 0.2 mN/m to 32.46 ± 0.01 mN/m, and the interfacial tension (IFT) of oil/ water from 20.45 ± 0.3 mN/m to 2.02 ± 0.22 mN/m. Similarly, the dynamic interfacial tension showed a reduction by two orders of magnitude, i.e., from 20.63 to 0.454 mN/m. Contact angle (CA) revealed that the tested solution has a good potential to modify rock wettability from oil-wet (120°) to more intermediate-wet (70.6°), which was in line with Amott test results. In addition, core flooding resulted in an additional recovery fall of 4.6 to 8.9% of the oil volume initially in place. It was found that incremental oil recovery was mainly due to wettability alteration toward intermediate wet coupled with IFT reduction. Moreover, the anionic biosurfactant exhibited good thermal stability after 6 months of aging at 70 °C, as indicated by stable surface/interfacial tension, contact angle, and recovery factors. Results of this study support the potential of this biosurfactant for EOR applications in carbonate reservoirs.

## 1 Introduction

Recently, the world’s energy consumption depends mainly on fossil fuels, accounting for as high as 81% of the total energy (Rapier, 2020) and the demand is expected to increase by 53% between now and 2030 (Sheng, 2013). Economically, it is also noteworthy that, in the Middle East, ~37–77% of government revenues come from the hydrocarbon sector (Energy Information Administration, 2016; Barnet et al., 2025). Therefore, expanding oil production to meet the huge demand cannot be achieved by relying only on conventional oil recovery (primary and secondary), where the maximum recovery that can be extracted from it is no more than 40–45% (Sen, 2008; Elshafie et al., 2015; Singh et al., 2025). Thus, enhanced oil recovery (EOR) techniques are strongly needed. Sheng (2013), with reference to market statistical analysis done by British Petroleum (BP), reported that 61% of the world’s conventional oil reserves are located in the Middle East, where approximately 70% of these reserves are in carbonate reservoirs (Schlumberger, 2007). Moreover, there are approximately 3,000 billion barrels of oil and 3,000 trillion standard cubic feet (SCF) of gas remaining in place in carbonates (Sheng, 2013). As a result, the importance of carbonate reservoirs has become obvious compared to other types of reserves and will increase dramatically in the near future. There are some reported advanced industrial process monitoring methods based on a dynamic machine learning approach for such physio-chemical analysis processes (Ali et al., 2024, 2025a, 2025b, 2026).

In spite of this great potential, improving oil recovery in tight carbonate reservoirs is not an easy task due to technical and economic challenges. This was clearly demonstrated by Alvarado and Manrique (2010), who found that only 18% of 1,507 international EOR applications, which were focused mainly on thermal and chemical projects, were performed in carbonate reservoirs, compared to 78% in sandstone reservoirs, during the last decade. There are several studies that cover the complex interplays of factors such as petroleum production chemistry, emulsion behavior, integrated flow-assurance strategies, surfactant performance, demulsification mechanisms, and combined chemical treatment approaches relevant to oilfield operations (Khormali, 2021; Khormali, 2023; Soroush and Azizollah, 2025).

Economic considerations have emerged as a critical challenge within the petroleum industry, particularly in recent years, as the sector has faced pronounced volatility in oil prices, marked by sharp declines to historic lows and intermittent surges to temporary peaks, as global markets react to geopolitical issues. Because of that, microbial enhanced oil recovery (MEOR) has become the most exciting topic in energy research as an environmentally friendly and low-operating-cost technology (Safdel et al., 2017). Although this technology has a significant positive impact on enhancing oil recovery from both sandstone and carbonate reservoirs, it is still widely underinvested (Safdel et al., 2017; Alkan et al., 2022; Ke et al., 2024; Li et al., 2025; Liu et al., 2026). Several studies highlighted potential *in situ* or *ex situ* microbial applications for enhancing oil recovery from carbonate reservoirs (Haddad et al., 2023; Ghorbani et al., 2024; Kapse et al., 2024; Sokolova et al., 2024).

Biosurfactants are biologically produced surfactants, which are reported to be either at par or even better at times as compared to chemical surfactants (Elshafie et al., 2015; Singh et al., 2025). In MEOR, the activity of biosurfactant-producing microorganisms facilitates improved oil displacement by reducing interfacial tension and altering reservoir wettability (Elshafie et al., 2015; Imanivarnosfaderani et al., 2022; Yu et al., 2023; Shabani et al., 2024; Shaikhah et al., 2024; Singh et al., 2025; Awadh et al., 2026). Accordingly, this study aimed to test the potential of glycolipid biosurfactant—sophorolipids, considered one of the most attractive microbial products for their lower cost, lower toxicity, and high stability. One of the Omani tight carbonate reservoirs characterized by high temperature and high salinity conditions was investigated by testing the compatibility of biosurfactant with field brine; investigating its effect on IFT at different concentrations; examining the effect of the biosurfactant on wettability alteration; testing its potential to enhance oil recovery via core-flooding experiments; and checking the stability of biosurfactant at reservoir conditions. The current study highlights the application of sophorolipids—microbial biosurfactants for enhanced oil recovery in carbonate reservoirs.

## 2 Materials and methods

### 2.1 Rock, fluids, and biosurfactant

#### 2.1.1 Rock samples

The rock core samples used in this study were obtained from an Omani carbonate reservoir. It is essential to clean the core plugs prior to proceeding with any experiments that require using the core, especially for the contact angle, Amott test, and core flooding experiments, to mimic the reservoir by achieving the water-wet state before oil migration. Soxhlet extraction process was used in this study using a mixture of extraction solvent that consisted mainly of 75% chloroform and 25% methanol. The principle of this process is based on the evaporation and condensation of the solvent. In this process, the condensed solvent passes through the core plugs, removing all the oil and any other substance before starting another cycle. The process was repeated until a clear color of the solvent was observed. After cleaning, the core samples were dried in an oven for 24 h for use. A detailed explanation of the procedure has been described elsewhere (Ukwungwu, 2017). The rock properties, which include gas porosity and permeability, were measured using a helium porosimeter (Vinci Technologies, France) and a gas permeability tester (Vinci Technologies, France). The average gas porosity and permeability of the samples are 28.89% and 4.54 mD, respectively. Table 1 shows the properties of the core plugs used.

**Table 1:** Properties of the core plugs used in this study.

Core #	Length (cm)	Diameter (cm)	Gas Porosity (%)	Gas Permeability (mD)	Experiment type
1	6.50	3.803	33.16	6.64	Core flooding
2	6.60	3.77	32.82	6.01	Core flooding
3	6.611	3.855	33.23	6.02	Core flooding
4	6.449	3.792	32.26	6.79	Core flooding
5	6.61	3.83	20.93	2.11	Core flooding
6	6.62	3.79	27.93	4.07	Amott
7	6.611	3.855	28.94	6.02	Amott
8	6.582	3.789	31.05	3.30	Amott
9	6.64	3.808	25.41	2.29	Stability
10	6.653	3.79	24.14	1.05	Stability
18	6.56	3.79	23.20	1.05	Core flooding after stability
19	6.45	3.81	24.62	2.30	Core flooding after stability

#### 2.1.2 Rock sample mineralogy analysis

X-ray diffraction (XRD) and X-ray fluorescence (XRF) analyses of the five representative core plugs were conducted with the assistance of the Central Analytical and Applied Research Unit (CAARU, College of Science, Sultan Qaboos University). Samples were prepared for XRD and XRF analyses as follows. First, the core slices were cleaned from the oil by aging overnight in acetone, followed by drying them in an oven. Then they were crushed with a pestle. Later, an HSM 100 manually operated pulverizer milling machine was used to convert them into powder, grain size 14 μm. The XRD pattern of the powders was measured using a PANalytical X’Pert PRO X-ray diffraction unit, compared with a standard pattern to identify the sample phases.

#### 2.1.3 Crude oil

The crude oil was brought directly from the field of interest and contained both water and some impurities. Core pores must be protected from plugging in by impurities. Therefore, the crude oil was separated from water by gravity at reservoir temperature (70 °C). Oil properties, which include viscosity, density, and API, were then measured using a viscosity meter (Anton Paar, Austria) and a density meter (Anton Paar, Austria).

#### 2.1.4 Formation water/brine

Filtered formation water samples were analyzed at the Central Analytical and Applied Research Unit (CAARU, College of Science, Sultan Qaboos University) for total water chemistry using inductively coupled plasma-OES (ICP-OES) (Perkin Elmer, Massachusetts, United States) and ion chromatography (IC) (Metrohm, Herisau, Switzerland). The conductivity was analyzed at room temperature using an electric conductivity meter. Then the sample was acid-digested with HNO_3_ for ICP analysis. After that, they were filtered through 0.45 *μ* filters and diluted properly prior to IC analysis.

#### 2.1.5 Biosurfactant

The anionic biosurfactant used in this study—sophorolipids was produced as reported before (Elshafie et al., 2015; Joshi et al., 2022; Singh et al., 2025). The formation water, the biosurfactant, and the crude oil samples used in this research were filtered as described above.

##### 2.1.5.1 Surface tension and interfacial tension measurements

Initially, surface tension (ST) and interfacial tension (IFT) measurements were performed using the drop-collapse method (Pendent Drop Tensiometer, Kruss, Germany) at room temperature. Different concentrations of biosurfactant were used for the analysis. For IFT measurements using the drop-collapse method, hexadecane was used as the lighter, hydrophobic phase. Since reducing the interfacial tension of the liquid interface is one of the most attractive features of biosurfactants, this characteristic was investigated at reservoir temperature to obtain more realistic and representative values. This experiment was conducted using a spinning drop tensiometer (SDT, Hamburg, Germany). The procedure was initiated by injecting a drop of the light phase (crude oil) into 1 mL of the heavy phase (aqueous solution) in a capillary tube, followed by loading the tube into the instrument and adjusting it so the drop would be placed in the middle. The glass tube was spun, and the drop was deformed by a radial pressure gradient, which caused it to elongate. IFT will oppose this elongation; hence, the greater the elongation, the lower the IFT will be recorded, and vice versa. After the drop stabilized, the IFT was measured from the oil drop geometry using Young–Laplace models, where minimum IFT is preferred to optimize the recovery process (Al-Ghailani et al., 2020).

##### 2.1.5.2 Critical micelle concentration determination

Surfactant properties are observed to change abruptly at a point called critical micelle concentration (CMC), where surfactant molecules self-aggregate into spherical structures known as micelles, which contain 50 or more surfactant molecules. In field applications, surfactants are used above their CMC value to avoid surfactant absorption. Another application of CMC is to determine the efficiency of the biosurfactant and allows the comparison between different surfactants, as the lower CMC means reaching a lower IFT; thus, a lesser amount of surfactant is required (Najimi et al., 2020). To determine CMC, ST and IFT measurements were carried out for nine biosurfactant solutions at different concentrations by the pendent drop method using a Kruss Drop Shape Analyzer (DSA100, Germany). To measure ST, a drop of the sample (biosurfactant solution) was produced with a syringe and placed in air. Similarly, for the IFT, a drop of the sample, the heavy phase, was immersed in n-hexadecane, the surrounding light phase. The image was then acquired and analyzed with the associated ADVANCE™ software. The measurements were made at ambient temperature (25 ± 2 °C) and a pressure of 1 ATM, and the average of three readings is reported for each of the three independent experiments (Adibhatla, 2006).

### 2.2 Contact angle measurements

**Figure 1:**
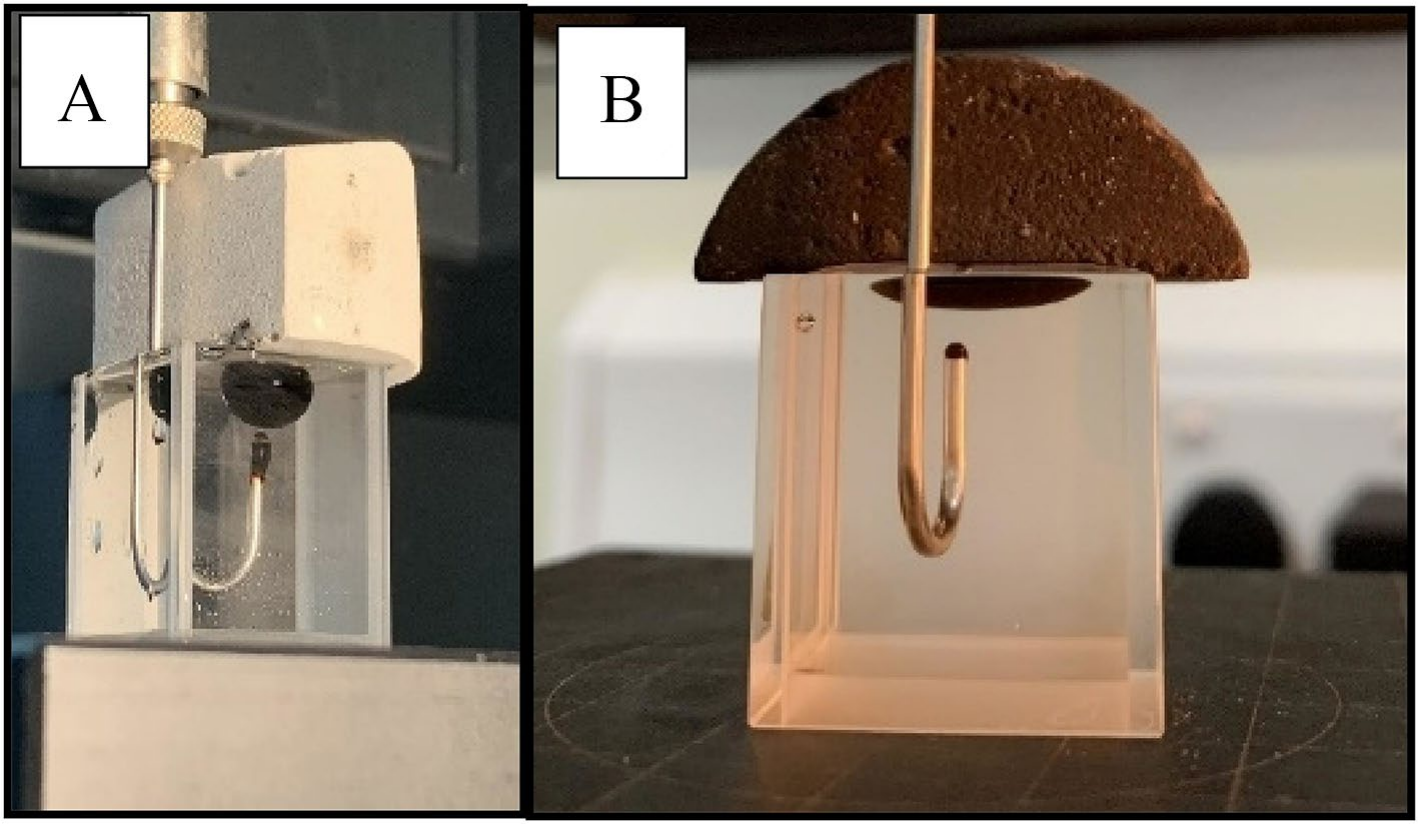
Cross-sectional view of crude oil drops on carbonate core surfaces: **(A)** Before aging in crude oil and **(B)** After aging in crude oil.

The Drop Shape Analysis system DSA 100 (KRUSS, Germany) was again used through the captive bubble method in order to measure the contact angle (CA) to have a good understanding of reservoir rock preferential-wetting and examine the biosurfactant ability to alter the rock wetting tendency. The measurement was conducted by cutting cylindrical discs from the carbonate core for use as rock surfaces. The discs were polished with sandpaper to avoid hysteresis with respect to surface roughness. Then, the discs were initially incubated in oil for 2 weeks at reservoir temperature (70 °C) to restore their original wettability. After that, the discs were incubated in different concentrations of biosurfactant—0.0625, 0.125, 0.25, 0.5, 1, 2, and 3% for a week at the same temperature, where one disc was incubated in the formation brine to act as a control (reference point). After aging, the carbonate discs were placed on top of a glass cube filled with brine that wetted their surfaces. A droplet of oil was placed under the disc surface by injecting it inside the brine using a J-shaped needle, and the picture was taken immediately by the camera and analyzed by the associated software for contact angle measurement (Chen et al., 2018). The CA was measured under ambient conditions (25 °C and 1 atm) in three phases: a droplet of crude oil, the carbonate rock surface, and the formation brine. The reported CA is an average of at least three independent measurements, including any surface heterogeneities. The cross-sectional view of oil drops on carbonate discs before and after aging in oil was recorded (Figure 1).

The system is considered water-wet if *θ* lies between 0 and 70°. The contact angle of an oil–wet system is within the range of 115° to 180°. What is in between is referred to as intermediate-wet; hence, θ is from 70° to 115° (Schön, 2011).

### 2.3 Amott wettability test

It was necessary to further study the effect of some of the selected biosurfactant solutions on the rock wettability alteration using another accurate methodology, the Amott test. Three experiments were conducted to test biosurfactants at 1 and 2% (v/v), in addition to the previously introduced reference test. The procedure for determining the wettability index was as follows: First, the core was saturated with brine and kept in a dissector for 24 h, then loaded into the core holder and flooded with brine. Then, the core was flooded with oil and aged in it for a month to restore its original wettability. The core was then loaded into an Amott cell filled with formation water or biosurfactant solutions and left for 3 weeks to undergo spontaneous imbibition. The oil volume displaced by spontaneous imbibition was recorded as (Vosp). Then, water or biosurfactant flooding at a rate of 0.4 cc/min was applied for 12 h for the forced imbibition process. The oil volume displaced by forced water/biosurfactant imbibition was recorded as (Vof). The core was then loaded into an inverted Amott cell filled with oil and left for 3 weeks to undergo spontaneous imbibition. Water or biosurfactant volume displaced by oil spontaneous imbibition was recorded as (Vwsp). Then, oil flooding at a rate of 0.4 cc/min was conducted for 12 h for the forced imbibition process. Water/biosurfactant displaced volume by oil forced imbibition was recorded as (Vwf). Finally, the Amott index (*WI*) was calculated by differentiating the oil wettability index (*OWI*) from the water/biosurfactant wettability index (*WWI*) as follows [Equations 1–3]:


WI=WWI−OWI (1)

Where, WI = Amott index, WWI = water/biosurfactant wettability index, and OWI = oil wettability index.

Where the water/biosurfactant and oil wettability indexes are given by:


$$\mathrm{WWI}=\frac{\text{Vosp}}{\text{Vosp}+\mathrm{Vof}}$$ (2)

Where, Vosp = oil volume displaced by spontaneous imbibition and Vof = oil volume displaced by forced water/biosurfactant imbibition.


$$\mathrm{OWI}=\frac{\text{Vwsp}}{\text{Vwsp}+\mathrm{Vwf}}$$ (3)

Where, Vwsp = water or biosurfactant volume displaced by oil spontaneous imbibitionand Vwf = water/biosurfactant displaced volume by oil forced imbibition.

A wettability index (*WI*) of (−1 to −0.3) indicates an oil–wet system, (−0.3 to +0.3) indicates an intermediate-wet, and (+0.3 to 1) indicates a water–wet system (Lucia et al., 2003).

### 2.4 Core flooding experiments

**Figure 2:**
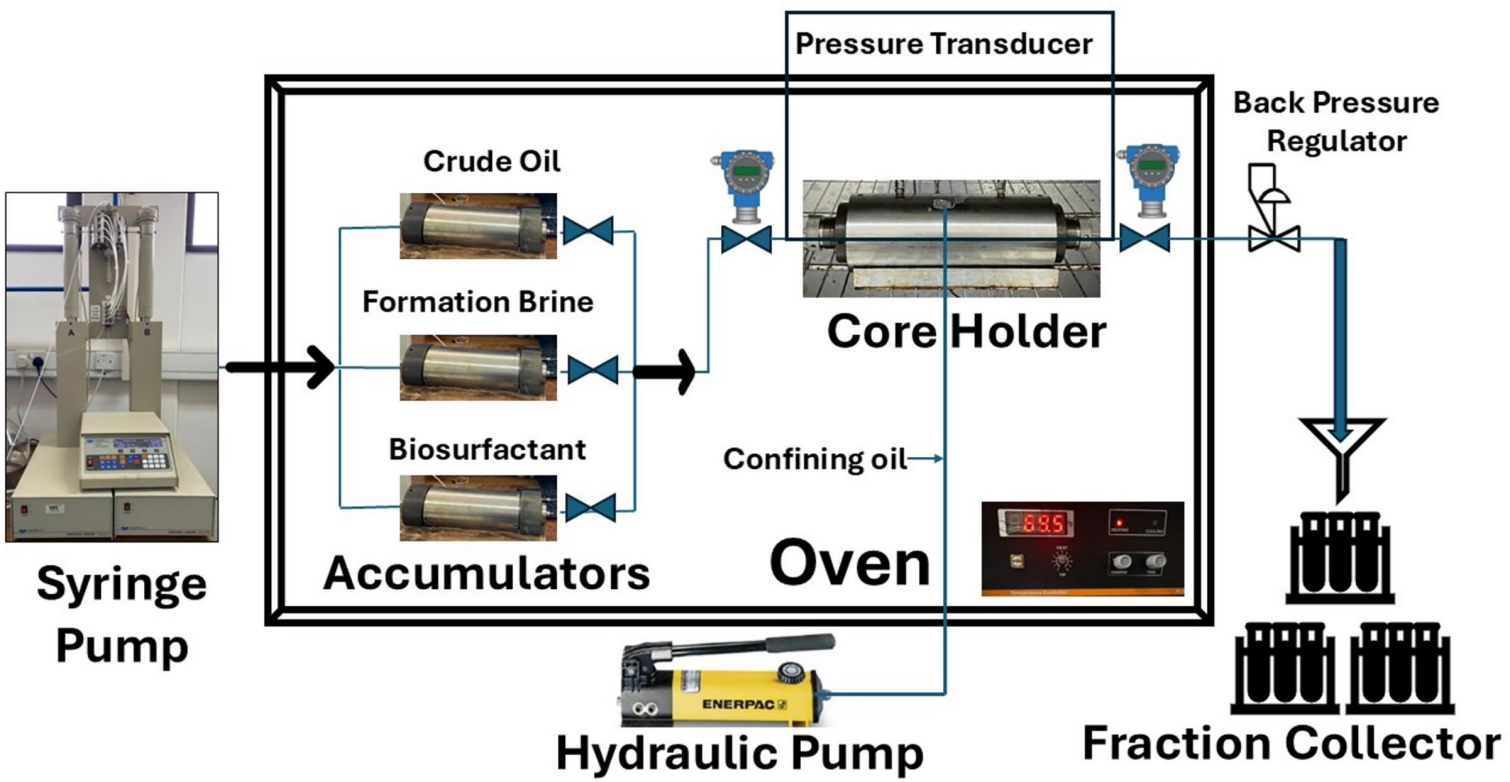
Core-flooding apparatus used for experiments at reservoir temperature.

Laboratory core floods were the primary component of this research to demonstrate oil recovery efficiency at each bio-based surfactant concentration. Initially, each core was saturated to 100% with reservoir brine by immersing it in a filled cup in a vacuum desiccator for 24 h, and the pore volume was calculated as the net change between the dry and wet weights. The saturated core was loaded into the core holder and connected to the core flooding rig lines, and a confining pressure of 1,000 psi was applied. The liquid permeability of the core was calculated by flooding it with brine at different rates. The core was then displaced by reservoir oil until no water came out, to establish the initial oil saturation. The oil initially in place (OIIP) was determined by the volume of water that was displaced by oil, taking into consideration the dead volume of the system; the initial oil saturation (Soi) was determined by dividing the OIIP over PV. The initial water saturation (Swi) was determined by subtracting Soi from unity. To obtain oil recovery by water flooding, the core was injected with brine at a constant rate of 0.4 cc/min until no remaining oil was produced, and the volume of oil produced was recorded gradually over time. The water flood recovery factor (WF RF) and residual oil saturation (Sor) were then determined. Injection into the core was continued with the biosurfactant until no more oil came out. The volumes of oil produced and the differential pressure readings were recorded manually with time, with the entire process conducted at 70 °C to mimic the reservoir temperature. The illustration below is a core-flooding set-up used in this experiment (Figure 2; Al-Ghailani et al., 2020).

### 2.5 Biosurfactant thermal stability studies

To ensure the activity of the tested biosurfactant to induce oil recovery over a long period, a thermal stability test was performed in which two selected biosurfactant solutions of 1 and 2% were aged at a reservoir temperature of 70 °C for 6 months, and their efficiency was tested by ST and IFT monthly after preparation. In addition, wettability alteration was also measured by contact angle, and further examinations were conducted by core flooding at the end of the mentioned period.

## 3 Results and discussion

### 3.1 Reservoir mineralogy and fluid analysis

**Figure 3:**
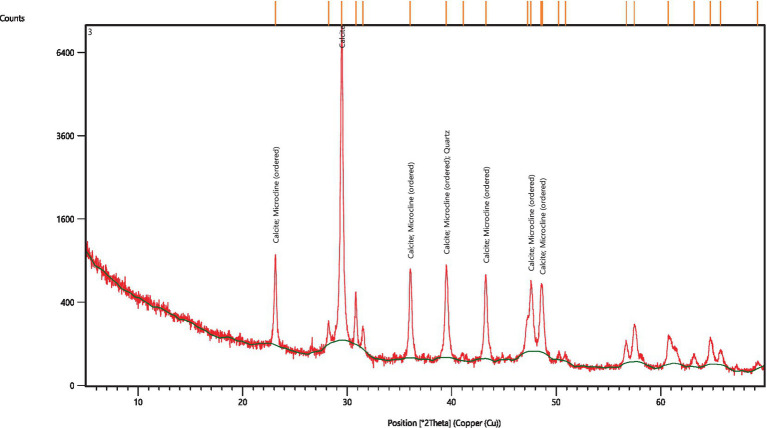
XRD analysis of major minerals from the core plug sample.

The XRD qualitative and quantitative analyses of the reservoir core samples are presented in Figure 3. The results revealed that the major component was calcite (90%), with minor amounts of microcline (<5%) and quartz (<5%). The XRF analysis for the major and trace elements of the samples showed (concentration in ppm): Mn, 70.8; Sr., 424.1; Te, 61.5; Ba, 26.9; Cr, 6.7; Br, 7.1; La, 5.9, and the rest of the elements were present at <5 ppm.

The crude oil provided by the local oil company had: density (g/cc) @25 °C, 0.91117; density (g/cc) @70 °C, 0.88118; viscosity (cp), @70 °C, 12.2; API gravity, 23.8°. The formation water was analyzed by ICP-MS and IC for water chemistry analysis. The result showed that the total dissolved salts were 227,000 ppm with 24,927 ppm of hardness. The major anions and cations were (ppm): fluoride, 5.641; chloride, 130928.46; bromide, 890.25; nitrate, 34.25; sulphate, 37.55; lithium, 18.781; sodium, 69233.29; potassium, 970.73; calcium, 18848.37; magnesium, 6079.45; Ba, 8.84; Fe, 5.20; B, 54.56; Si, 7.45; Sr., 735.12.

### 3.2 Compatibility test

**Figure 4:**
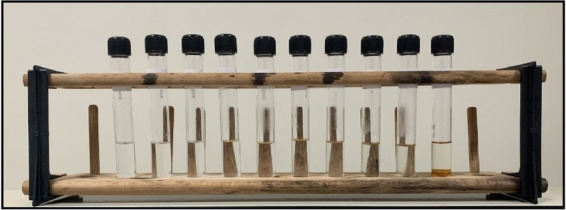
Compatibility test of different biosurfactant concentrations in formation water/brine: Brine, biosurfactants: 0.0625, 0.125, 0.25, 0.5, 1, 2, 3, 5, and 10% (v/v), from left to right after incubation at 70 °C for 72 h.

For this test, a series of nine tubes with different biosurfactant concentrations (0.0625, 0.125, 0.25, 0.5, 1, 2, 3, 5, and 10%) (v/v) were dissolved in formation water. The samples were visually observed at reservoir temperature (70 °C) after 3 days of incubation. The analysis showed that biosurfactant is compatible with field brine as it exhibited clear behavior, and no precipitation was observed up to 5% (Figure 4). Testing the compatibility of the biosurfactant with formation water is very important to avoid incompatibility issues, such as suspended solids or precipitation, which can block the pore system and cause injectivity problems. (Liu, 2011) reported that it is necessary to inject a single-phase clear surfactant solution as a flooding process, as a hazy surfactant solution was proven to contribute to low oil recovery.

### 3.3 ST, IFT and CMC determination

**Figure 5:**
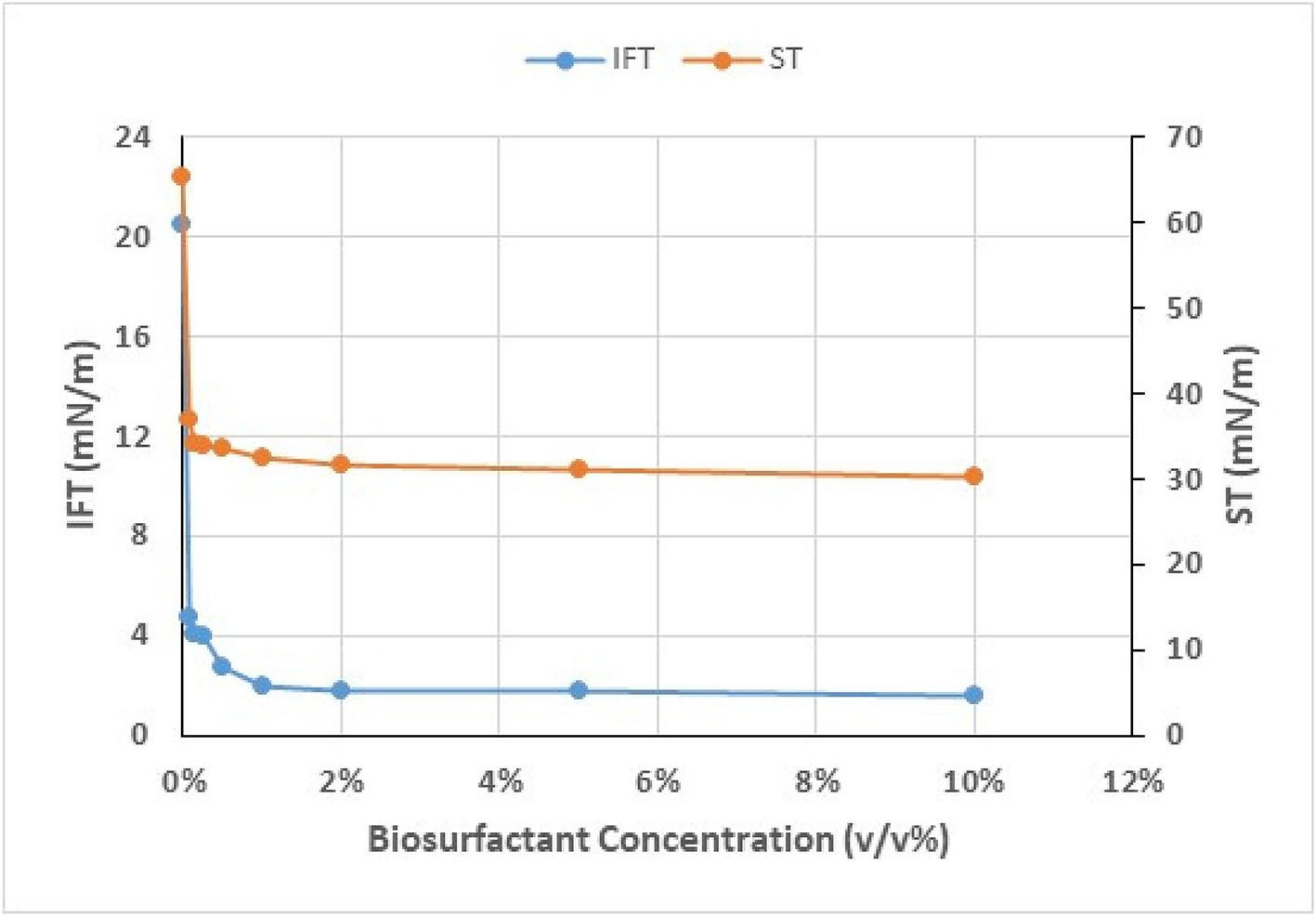
Surface tension (ST) and interfacial tension (IFT) (against hexadecane) analyses of biosurfactant solution with variable concentrations for CMC determination at room temperature.

Surface tension (ST) and interfacial tension (IFT) profiles of the biosurfactant as a function of concentration were plotted (Figure 5). ST and IFT decrease progressively as the biosurfactant concentration increases up to CMC, at which the profiles start to level off, and no significant change in values was noticed beyond that. ST decreased from an initial value of 65.3 ± 0.2 mN/m to 32.46 ± 0.13 mN/m at CMC of 1%. Similarly, the IFT decreased from 20.45 ± 0.3 mN/m to 2.02 ± 0.12 mN/m at the same point. The obtained CMC value falls within the range of other biosurfactants reported previously, considering 1% for *C. lipolytica* biosurfactant grown in refinery waste (Rufino et al., 2007) and 2.5% for *C. glabrata* (Sarubbo et al., 2007). Singh et al. (2025) reported sophorolipid stability and IFT of 2.18 mN/m at 250 ppm concentration, under CO_2_-enriched conditions. Widiyaningsih et al. (2026) reported that a 0.5% sophorolipid solution reduced IFT and increased the water-wetness of the carbonate rock surface at 2000 ppm Ca^2+^ and Mg^2+^ ions, on Indiana limestone samples.

### 3.4 Interfacial tension

**Figure 6:**
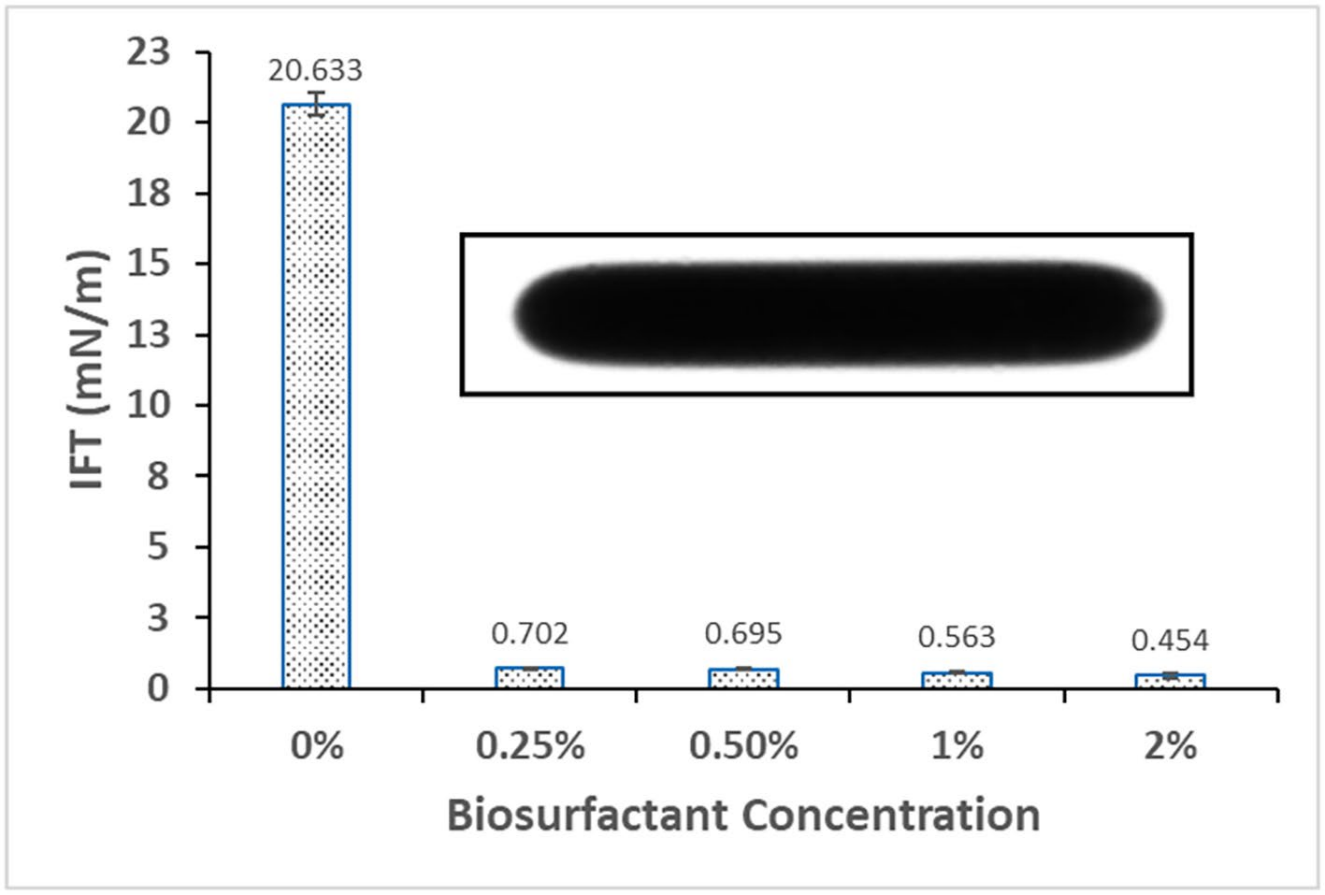
Snapshot of crude oil drop inside the capillary syringe in the presence of 2% biosurfactant, and interfacial tension (IFT) analysis of different biosurfactant concentrations at reservoir temperature (70 °C) using spinning drop tensiometer.

The dynamic IFT between the biosurfactant and crude oil was measured using a spinning drop tensiometer at reservoir temperature, and a snapshot of a crude oil drop inside the capillary syringe in the presence of 2% biosurfactant from the spinning drop tensiometer was obtained (Figure 6). It was found that the IFT of the prepared solutions reduced slightly with time until they stabilized. The IFT decreased by two orders of magnitude from 20.63 to 0.454 mN/m with a reduction of ~97% (Figure 6). Generally, the spinning drop tensiometer measures the dynamic interfacial tension; therefore, the measured values are relatively lower than those obtained by the static approach measured by the pendent drop. Moreover, the static IFT was conducted against hexadecane, which may lead to higher interfacial tension, as it does not contain acids that contribute to IFT reduction (Souayeh et al., 2014). It is well known in the literature that, for efficient displacement of residual oil, IFT needs to reach an ultra-low value, reducing by three orders of magnitude, which can be challenging and expensive for both chemical surfactants and biosurfactants (Hajibagheri et al., 2017). Nevertheless, several studies have shown that major types of biosurfactants have a restricted capability of reducing oil/water interfacial tension by up to 1 order of magnitude, which still has been found to have potential effects on oil recovery efficiency (Desai and Banat, 1997; Kryachko et al., 2013; Rabiei et al., 2013). In our case, we also observed that sophorolipids reduced the IFT against crude oil by two orders of magnitude, and later further confirmed the potential role in oil recovery from carbonate core plugs under reservoir conditions.

### 3.5 Contact angle

**Figure 7:**
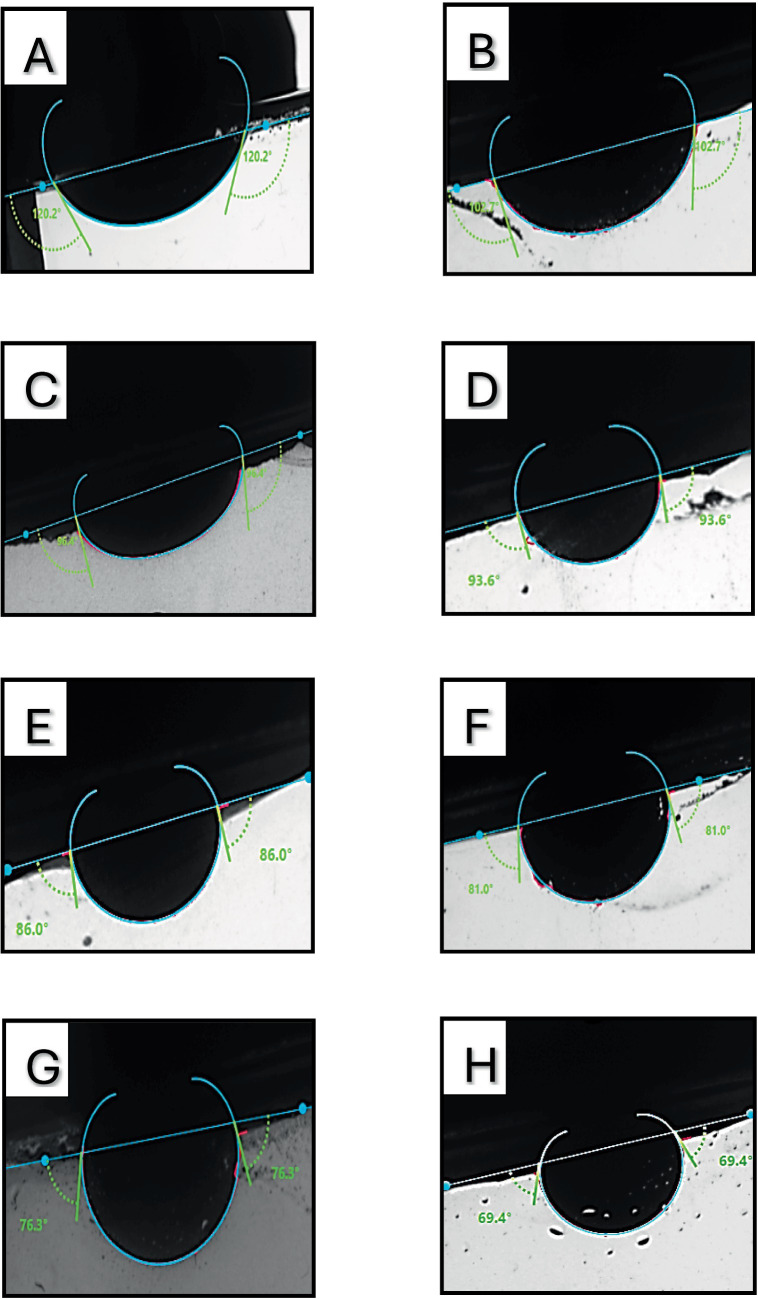
Images of contact angle for core slices initially aged in crude oil for 2 weeks followed by incubation in brine **(A)**, 0.0625% **(B)**, 0.125% **(C)**, 0.25% **(D)**, 0.5% **(E)**, 1% **(F)**, 2% **(G)**, and 3% **(H)** biosurfactant for a week.

The technique of contact angle measurement was utilized to give a quantitative indication of the wetting affinity of reservoir rock surfaces aged at different biosurfactant concentrations toward crude oil (Figure 7). It was found that the contact angle of formation water changed from an initial value of 120° ± 0.42 to 108.8° ± 4.3 when treated with 0.0625%. It was also found that the contact angle continued to decrease slightly, reaching 103.4° ± 4.6 and 99.5° ± 4.1, respectively, upon increasing the concentration to 0.125 and 0.25%. A significant drop in CA was noticed between 0.25 and 2%, where the CMC of the biosurfactant existed. In addition, it was noticed that the curve stabilized at the higher tested concentration (3%) with an additional decrease of only approximately 2.3°. The overall reduction in CA was approximately 41%. The biosurfactant changed the wetting tendency of the carbonate surface from weakly oil-wet to intermediate water-wet when incubated in biosurfactant at concentrations of 0.125, 0.25, 0.5, 1, 2, and 3% (Figure 7; Table 2). The maximum standard deviation of the measurements was ±4°, which is comparable to other studies (Hajibagheri et al., 2017).

**Table 2:** Contact angle analysis summary, using different biosurfactant concentrations.

Test	Contact angle	Wetting behavior
Brine	120° ± 0.42	Oil-wet
0.0625%	108.8° ± 4.3	Oil-wet
0.125%	103.4° ± 4.6	Intermediate-wet
0.25%	99.5° ± 4.1	Intermediate-wet
0.5%	87.8° ± 1.3	Intermediate-wet
1%	79.4° ± 3.1	Intermediate-wet
2%	72.9° ± 2.8	Intermediate-wet
3%	70.6° ± 1.9	Intermediate-wet

A similar approach was achieved in a study conducted by Jarrahian et al. (2012) in which they tested the alteration of the contact angle of carbonate treated with 0.05, 0.1, 0.3, 2.5, and 3% of chemically based anionic surfactant (SDS). They reported that SDS can alter the wettability from 138° (oil-wet) to 74° (intermediate-wet) at 3% SDS, with a total reduction of 46%, which is close to the tested biosurfactant in this research. This result confirmed that the anionic bio-based surfactant is comparable to the chemical-based surfactant. Hajibagheri et al. (2017) found that the biosurfactant scored the highest reduction in contact angle from 138° to approximately 30° compared to 79° and approximately 34° of chemical anionic and cationic, respectively. They concluded that the bio-solution was the most effective at changing the wettability of carbonate rocks from a strongly oil-wet state toward a strongly water-wet state, followed by chemical cationic, which further supports the high capability of biosurfactants to alter the wettability of carbonate surfaces. Several papers highlighted different types of wettability modifiers for calcite/carbonate rock surfaces, such as surfactants, nanoparticles, salts, alkali, and biosurfactants (Deng et al., 2019; Abdulhadi and Ali, 2025; Bazyari et al., 2025; Chang et al., 2026). Those papers highlighted the important role of high temperatures, high salinity, rock properties (oil-wet, water-wet, or mixed-wet), geological formation, nature of surfactant (anionic or cationic), concentration of surfactant, surfactant stability, and the interplay of complex solution chemistry, which determines the optimum EOR flooding strategy.

### 3.6 Amott index

**Figure 8:**
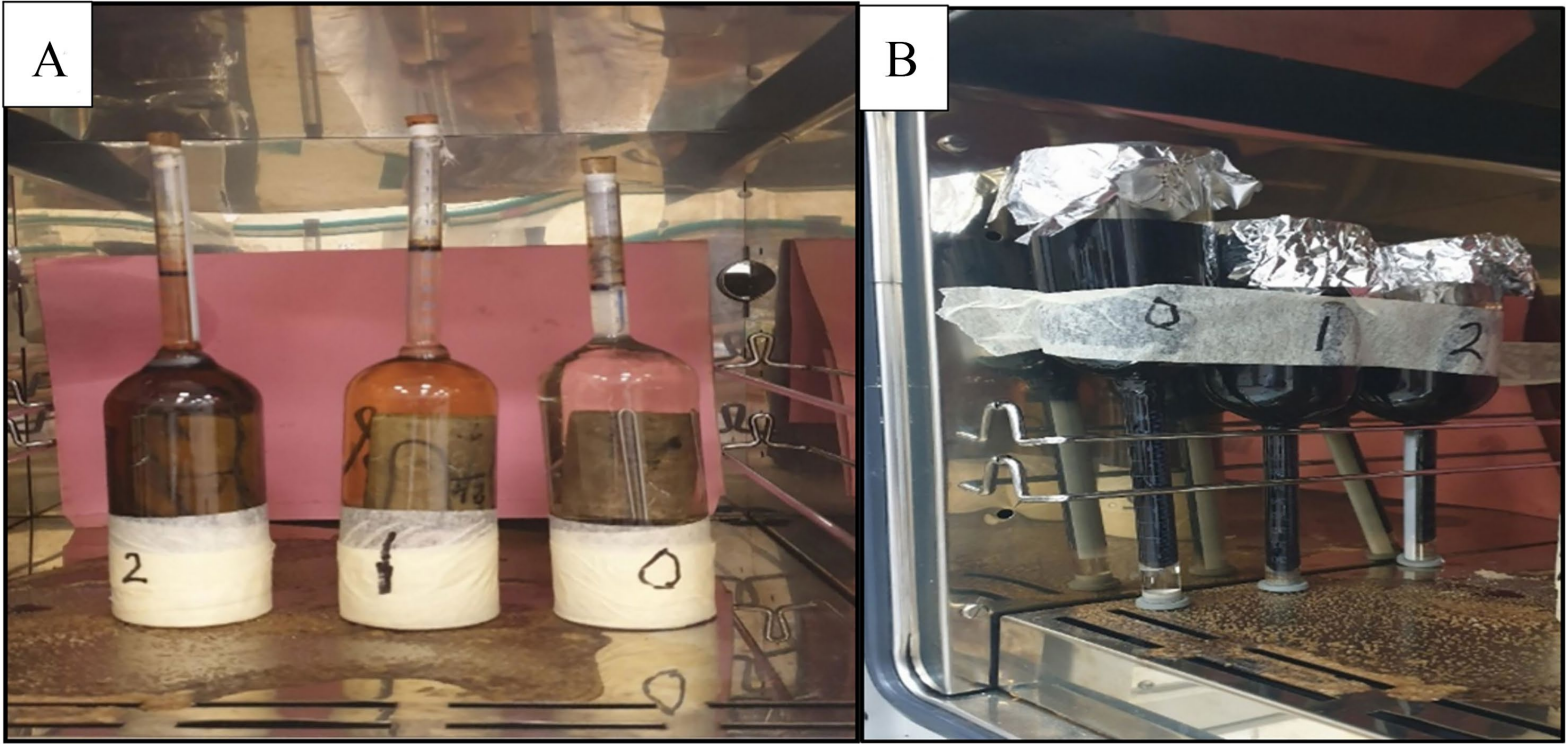
First stage of the Amott test **(A)** in which core plugs were aged in different concentrations of biosurfactant 0, 1, and 2% (v/v) for 3 weeks for the spontaneous imbibition process **(B)** Second stage of the Amott test, in which core plugs were aged in crude oil for 3 weeks for the spontaneous imbibition process.

The extent of the wetting alteration due to biosurfactant treatment was also examined by Amott tests as another more accurate quantitative method since the contact angle method has some limitations with respect to rock surface heterogeneity, and it has been found experimentally that a drop of liquid on a surface can have multi-stable contact angles (Anderson, 1986). Table 3 summarizes the results, where the control test (untreated sample) indicated that the cores were oil-wet, as the Amott wettability index (WI) was calculated to be approximately −0.354. It was found that the biosurfactant increased the WI at both concentrations, 1 and 2%, to 0.03 and 0.047, respectively. Amott test results are consistent with contact angle measurements, indicating wettability alteration from oil-wet toward intermediate-wet. Amott index has a number of advantages. For example, it is sensitive to mixed wettability (Anderson, 1986) and it is a well-developed methodology (Thyne, 2013). On the other hand, it has some drawbacks, such as being difficult to apply in low-quality core plugs (low porosity and permeability), being time-consuming, expensive, and labor-intensive. Figure 8 shows the first and second stages of the Amott test, in which core plugs were aged in biosurfactant solution and crude oil for 3 weeks, respectively. There are reports of biosurfactants with high or low hydrophile–lipophile balance (HLB) that could alter the wettability to either totally water-wet or neutral/intermediate-wet (Jahanbani Veshareh and Ayatollahi, 2020; Chang et al., 2026); in either case, the biosurfactant could enhance the oil recovery from carbonate reservoirs. Though it would be beneficial, it is not necessarily desirable to achieve complete water-wet status.

**Table 3:** Amott wettability index determination.

Test	Vo_sp_	Vo_f_	Vw_sp_	Vw_f_	WWI	OWI	WI	Wetting behavior
Control	0.67	5.49	2.67	3.11	0.108	0.462	−0.354	Oil-wet
1% BS	1.72	5.68	1.17	4.63	0.232	0.202	0.03	Intermediate-wet
2% BS	2.68	10.22	1.26	6.55	0.208	0.161	0.047	Intermediate-wet

### 3.7 Core flooding

**Figure 9:**
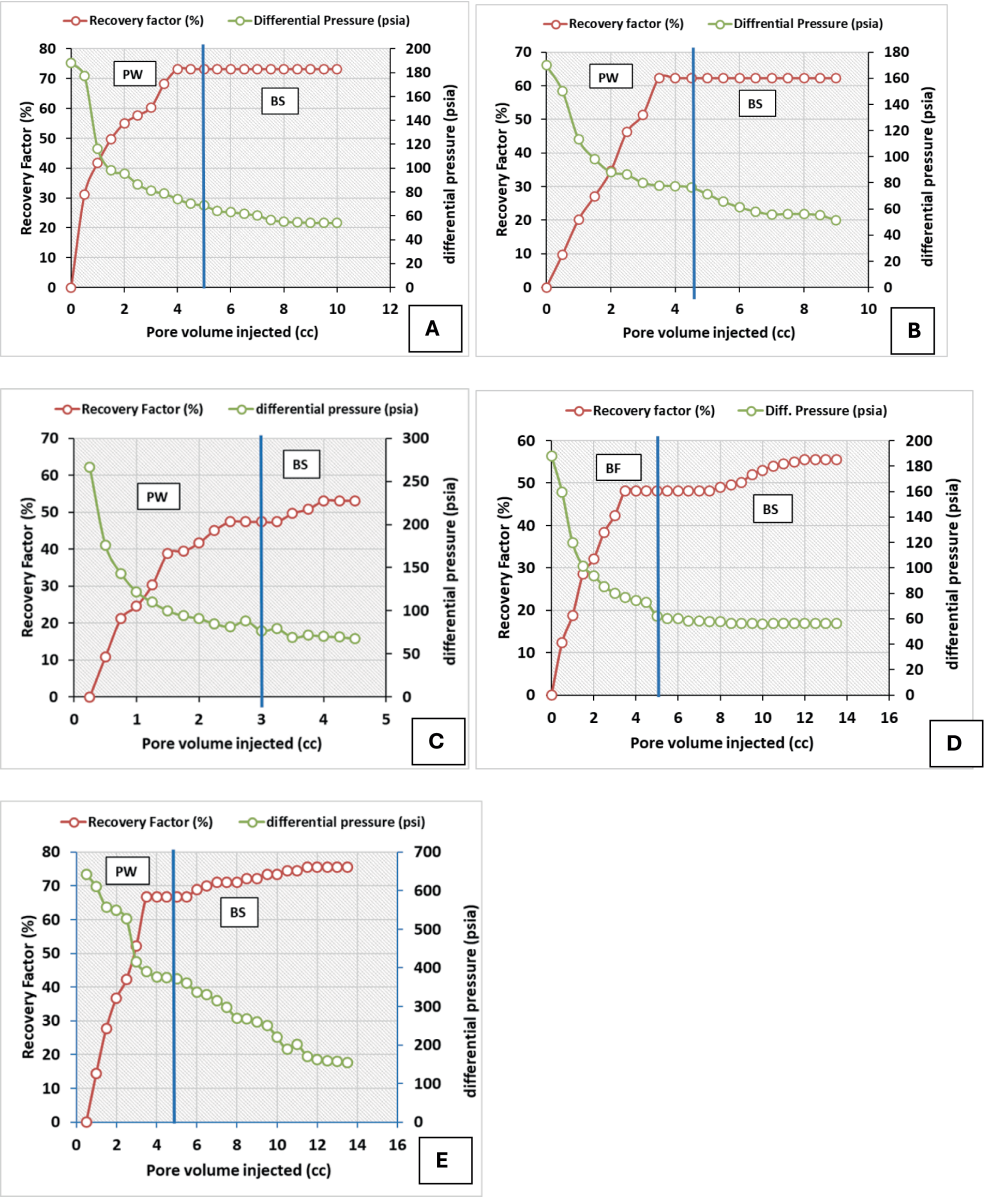
Core flooding experiments with **(A)** 0.0625% BS, **(B)** 0.5% BS, **(C)** 1% BS, **(D)** 2% BS, and **(E)** 3% biosurfactant in reservoir core plugs at 70 °C.

Core flooding experiments were conducted to investigate the performance of a biosurfactant to extract the trapped oil. Based on test results of ST, IFT, as well as the wettability alteration conducted by contact angle, five different biosurfactant concentrations were chosen for the core flooding experiment: 0.0625, 0.5, 1, 2, and 3%. The average area for these carbonate cores is 11.28 cm^2^; the average liquid porosity and permeability were found equal to 27.98% and 2.17 mD, respectively. The average pore volume (PV) was 20.57 cc, calculated from the averaged dry and wet weights (Table 4). The results, which are presented in Figures 9A–E, reveal that the initial oil saturation (Soi) was approximately 73%. In addition, secondary recovery (water flooding) produced an average of 59% after 5 PV were injected. After waterflooding, the residual oil saturation (Sor) was 30.1% on average. No extra oil production was observed by using lower concentrations of 0.0625 and 0.5%. This failure is possibly due to an insufficient amount of biosurfactant applied to the surface of the core sample, which resulted in complete adsorption of biosurfactant as reported by Yakimov et al. (1997) and Al-Bahry et al. (2013). Incremental oil recovery was observed when the concentration increased to 1, 2, and 3% BS. The microbial culture yielded oil production of 9.8, 12.6, and 26% of residual oil, corresponding to 4.6, 6.5, and 8.9% of the oil initially in place, respectively.

**Table 4:** Summary of core flooding experiments.

Experimental information	Core flood using different biosurfactant concentrations					Core flood after 6-month stability test	
BS concentration (%)	0.0625%	0.5%	1%	2%	3%	1%	2%
Core #	1	2	3	4	5	19	18
Porosity (%)	32.59%	29.93%	27.58%	31.64%	18.23%	24.62%	23.20%
Gas permeability (mD)	6.00	6.64	6.79	5.67	2.11	2.30	1.05
L (cm)	6.60	6.50	6.45	6.44	6.61	6.45	6.56
D (cm)	3.77	3.80	3.79	3.80	3.80	3.81	3.79
r (cm)	1.89	1.90	1.90	1.90	1.90	1.90	1.90
A (cm^2^)	11.16	11.35	11.29	11.33	11.35	11.38	11.28
Brine viscosity (cp)	0.61	0.61	0.61	0.61	0.61	0.61	0.61
Liquid permeability (mD)	2.69	2.78	2.37	2.36	0.63	0.85	0.31
Core dry weight (g)	131.90	126.20	129.00	135.30	155.48	153.00	156.70
Core wet weight (g)	155.80	148.20	149.00	158.30	169.10	171.00	173.80
Brine density (g/cm^3^)	1.00	1.00	1.00	1.00	1.00	1.00	1.00
PV (ml)	24.00	22.09	20.08	23.09	13.67	18.07	17.17
OIIP (ml)	18.9	14.4	17.5	15.4	9.0	12.8	10.2
Soi (%)	78.76	65.19	87.15	66.69	65.81	70.83	59.41
Swi (%)	21.24	34.81	12.85	33.31	34.19	29.17	40.59
Oil produced (ml)	11.40	9.60	9.3	7.40	6.00	6.70	5.60
Brine RF (%)	60.32	66.67	53.14	48.05	66.67	52.34	54.90
Residual Oil (ml)	7.50	4.80	8.2	8.00	3.00	6.1	4.6
Sor (%)	31.26	21.73	40.84	34.64	21.94	33.75	26.79
Eor (%) (out of OIIP)	0.00	0.00	4.6	6.49	8.89	7.0	12.75

These experiments are in line with IFT measurements where the curve stabilized at 1%, indicating the CMC of the solution at which the first production was observed. Contact angle results can also provide clear support, as they reveal that the wetting behavior has changed significantly, especially between 0.25 and 2%, in the direction of water wet. This implies that both mechanisms contributed to oil recovery. The combined effects of IFT reduction and wettability alteration using surfactants have been discussed in the literature (Kowalewski et al., 2006; Rabiei et al., 2013). Note that even though the highest EOR recovery percentage was obtained from the highest BS concentration, it still yielded the same oil recovery as 1% BS in terms of volume. This clearly refers to rock heterogeneity, since core#5 has the lowest porosity and permeability among the cores used in this experiment, which in turn resulted in lower PV, OIIP, and Sor. However, this biosurfactant still had the potential to maintain a recovery trend increasing as the concentration goes higher. A number of previous studies investigated the effectiveness of biosurfactant treatment in extracting trapped oil from porous media. Joshi et al. (2016) reported that biosurfactant produced by *Bacillus licheniformis* W16 resulted in a recovery of 24–26% over residual oil saturation. Al-Wahaibi et al. (2016) also reported a recovery of 6.8–18.5% of residual oil by injecting a biosurfactant produced by *Bacillus subtilis* following hot water injection. Both studies were performed on sandstone core plugs. While this study is similar to reports published by Al-Bahry et al. (2013) and Al-Hattali (2012), where they used carbonate core samples to investigate the potential of two types of biosurfactant produced by two different bacteria (*Bacillus subtilis* B20 and *B. licheniformis* W16) using Omani date molasses as a cheap carbon source required for biosurfactant production. Their studies yielded 9.7 and 28.5% residual oil, respectively. Widiyaningsih et al. (2026) reported approximately 28–32% recovery for different types of oil using sophorolipid injection. Singh et al. (2025) reported ~2.74–24.17% additional oil recovery using either sophorolipids alone or in combination with polymers.

### 3.8 Thermal stability

**Figure 10:**
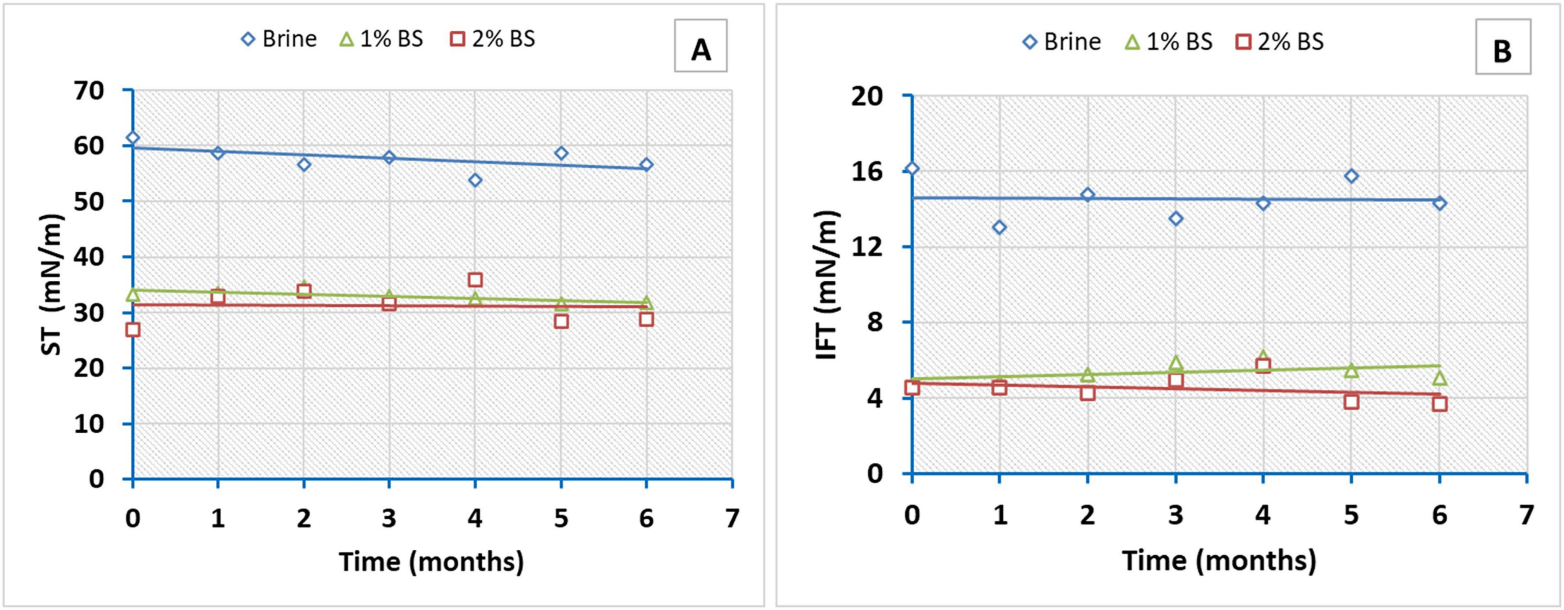
Effect of temperature on biosurfactant activity with respect to **(A)** surface tension and **(B)** interfacial tension. Both ST and IFT were analyzed for up to 6 months after incubating biosurfactant at 70 °C.

The biosurfactant showed high stability at a high reservoir temperature of 70 °C for up to 6 months. The ST started at 33.29 and ended at 31.73, recording an average value of 32.8 mN/m for 1% BS. Similarly, for 2% BS, the ST started at 30.6 to 28.78 mN/m, reaching an average of 31.2 mN/m (Figure 10). IFT against hexadecane also exhibited a stable trend for both concentrations. The measured values ranged from a minimum of 4.7 mN/m to a maximum of 6.1 mN/m, with an average IFT of 5.3 mN/m for 1% BS and from 3.6 to 5.7 mN/m, with an average value of 4.5 for 2% BS (Figure 10). The above results confirm the stability of the biosurfactants under extreme conditions. Al-Sulaimani et al. (2011) reported that after subjecting the biosurfactant to temperatures up to 80 °C for 20 days, no appreciable change was observed in ST and IFT. For instance, IFT ranged from 3.28 to 7.02 mN/m, with an average of 5.77 mN/m. Even after autoclaving at 120 °C for 15 min, their biosurfactant activity remained unaffected. Stability of biosurfactant under high temperature was also proven by Adhitya et al. (2019).

**Figure 11:**
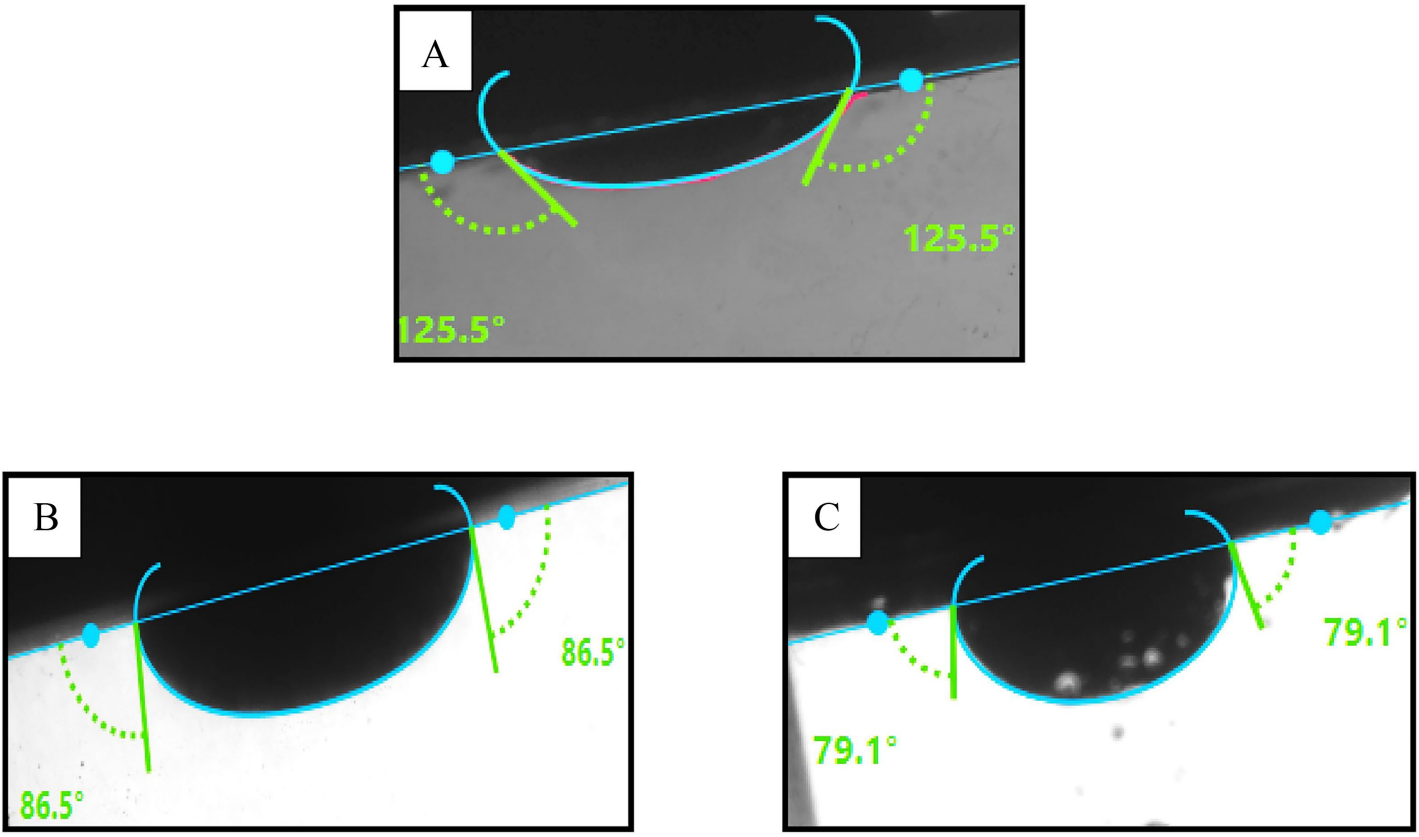
Contact angle measurements of brine **(A)**, biosurfactant 1% **(B)**, and biosurfactant 2% **(C)** after 6 months of incubation at 70 °C to test biosurfactant stability.

Contact angle results showed a negligible increase in values, consistent with the same wettability type reported previously (Figure 11; Table 5). Contact angle results are in line with ST/IFT test results, proving the stability of this biosurfactant.

**Table 5:** Summary of contact angle measurements—before and after 6 months of biosurfactant incubation at 70 °C.

Test	Contact angle		Wetting behavior
	Original test	Stability test (after 6 months of BS incubation at 70 °C)	
Brine	120° ± 0.42	125.9° ± 4.8	Oil wet
BS (1%)	79.4° ± 3.1	83.2° ± 2.3	Intermediate-wet
BS (2%)	72.9° ± 2.8	74.7° ± 3.3	Intermediate-wet

**Figure 12:**
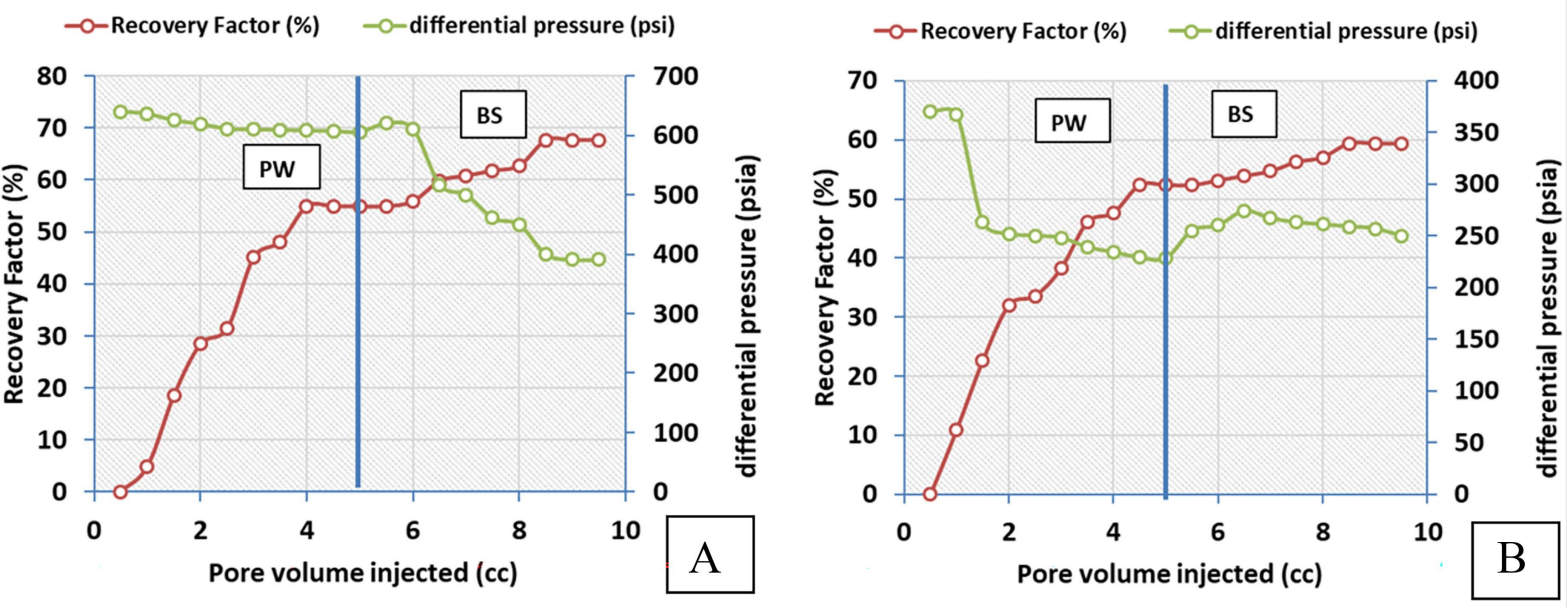
Core flooding experiments with **(A)** 1% BS and **(B)** 2% BS in reservoir core plugs after incubating the biosurfactants for 6 months at 70 °C.

Core flooding results also yielded similar EOR recovery factors, where 1% biosurfactant yielded 7% compared to the original recovery factor of 4.6% of OIIP (Figure 12A; Table 4). Similarly, 2% biosurfactant resulted in 12.7%, compared with an original recovery of 6.5% of OIIP (Figure 12B; Table 4). In general, the results obtained from this study agreed with the results reported previously. The 20-day biosurfactant stability was reported by Al-Sulaimani et al. (2011). However, the stability test in this research was extended to 6 months to include CA and core flooding studies. The results indicated that the biosurfactant was stable under the tested conditions.

## 4 Conclusion, future trends and recommendations

This study systematically evaluated the enhanced oil recovery (EOR) potential of an anionic sophorolipid biosurfactant for application in an Omani carbonate reservoir through experimental investigations such as mineralogical characterization, compatibility analysis, interfacial tension (IFT) and surface tension (ST) measurements, wettability assessment, spontaneous imbibition (Amott test), core flooding, and thermal stability evaluation. Mineralogical analysis confirmed that the reservoir rock is predominantly calcite, with minor amounts of microcline and quartz, thereby validating its classification as a carbonate reservoir. This is particularly important, as carbonate formations are typically oil-wet and challenging conventional EOR strategies. Compatibility tests demonstrated that the sophorolipid biosurfactant remains stable and effective in highly saline produced water, even at concentrations up to 5%, highlighting its robustness under harsh reservoir conditions. Static ST and IFT measurements established a critical micelle concentration (CMC) of 1% BS, beyond which no significant reduction in interfacial properties was observed. Dynamic IFT measurements revealed a substantial reduction in oil–water interfacial tension by two orders of magnitude, from approximately 20 mN/m to 0.454 mN/m at a reservoir temperature of 70 °C. This significant reduction enhances oil mobilization. Wettability studies further demonstrated a shift from strongly oil-wet to intermediate-wet conditions, as evidenced by both contact angle measurements and Amott indices, thereby confirming the dual functionality of the biosurfactant in altering rock-fluid interactions. Core flooding experiments showed incremental oil recovery ranging from 4.6% of oil initially in place (OIIP) at CMC concentration to a maximum of 8.9% at higher concentrations, indicating a clear concentration-dependent recovery behavior. Moreover, the 6-month thermal stability assessment confirmed that sophorolipids retained their physicochemical properties and EOR performance after prolonged exposure to elevated reservoir temperatures, demonstrating excellent long-term stability.

The novelty of the study lies in the demonstration of high salinity tolerance and long-term thermal stability (6 months), which is rarely reported together for sophorolipids, and the confirmation of simultaneous IFT reduction and wettability alteration by sophorolipids, key mechanisms for effective EOR in carbonates. In addition, it was also experimentally validated that after incubating sophorolipids for 6 months at reservoir temperature, they still showed EOR potential, making them a potential candidate for future field-scale studies. Overall, the findings establish sophorolipid biosurfactants as a promising, environmentally friendly alternative to conventional surfactants for EOR applications in carbonate reservoirs. However, there are still additional tests needed before it can be deployed on field scale applications: adsorption behavior on carbonate rock surfaces to quantify retention losses and optimize dosage requirements; injectivity tests using long core plugs or heterogeneous core systems to evaluate flow behavior, permeability impairment, and propagation efficiency in reservoir conditions; to explore synergistic combinations of chemical surfactants and sophorolipids for possible performance improvement and reduced cost.

## Data Availability

The original contributions presented in the study are included in the article/supplementary material, further inquiries can be directed to the corresponding author/s.
